# Efficacy and Safety of Corticosteroid Treatment in Patients With COVID-19: A Systematic Review and Meta-Analysis

**DOI:** 10.3389/fphar.2020.571156

**Published:** 2020-09-09

**Authors:** Wenwen Cheng, Yufeng Li, Liyan Cui, Ying Chen, Sharui Shan, Duan Xiao, Xiaoyun Chen, Zhuoming Chen, Anding Xu

**Affiliations:** ^1^Department of Neurology, The First Affiliated Hospital of Jinan University, Guangzhou, China; ^2^Department of Neurology and Stroke Center, The First Affiliated Hospital of Jinan University, Guangzhou, China; ^3^Department of Rehabilitation, The First Affiliated Hospital of Jinan University, Guangzhou, China; ^4^Department of Ideological and Political Theory Teaching, Maoming Polytechnic, Maoming, China; ^5^Department of Rehabilitation, The First Affiliated Hospital of Guangdong Pharmaceutical University, Guangzhou, China

**Keywords:** corticosteroid, COVID-19, SARS-CoV-2, efficacy, meta-analysis

## Abstract

**Background:**

COVID-19 is a type of pneumonia caused by severe acute respiratory syndrome coronavirus 2 (SARS-CoV-2) infection that was identified in December 2019. Corticosteroid therapy was empirically used for clinical treatment in the early stage of the disease outbreak; however, data regarding its efficacy and safety are controversial. The aim of this study was to evaluate the efficacy and safety of corticosteroid therapy in patients with COVID-19.

**Methods:**

The PubMed, Cochrane Library, EMBASE, Web of Science, China National Knowledge Infrastructure (CNKI), Wanfang, and China Science and Technology Journal (VIP) databases were searched for studies. Data on clinical improvement, mortality, virus clearance time, adverse events (AEs), utilization of mechanical ventilation, length of intensive care unit (ICU) hospitalization, and hospital stay were extracted by two authors independently. Study quality was assessed by the Newcastle Ottawa Scale (cohort studies). The pooled data were meta-analyzed using a random effects model, and the quality of evidence was rated using the GRADE approach.

**Results:**

Eleven cohort studies (corticosteroid group vs control group), two retrospective cohort studies (without control group), and seven case studies were identified. A total of 2840 patients were included. Compared with the control treatments, corticosteroid therapy was associated with clinical recovery (RR = 1.30, 95% CI [0.98, 1.72]) and a significantly shortened length of ICU hospitalization (RR = −6.50; 95% CI [−7.63 to −5.37]), but it did not affect the mortality ((RR = 1.59; 95% CI [0.69–3.66], *I^2^* = 93.5%), utilization of mechanical ventilation (RR = 0.35; 95% CI [0.10, 1.18]), duration of symptoms (WMD = 1.69; 95% CI [−0.24 to 3.62]) or virus clearance time (RR = 1.01; 95% CI [−0.91 to 2.92], *I^2^* = 57%) in COVID-19 patients. Treatment with corticosteroids in patients with COVID-19 may cause mild adverse outcomes. The quality of evidence was low or very low for all outcomes.

**Conclusion:**

The findings of our study indicate that corticosteroid therapy is not highly effective, but it appears to improve prognosis and promote clinical recovery in patients with severe COVID-19.

## Introduction

Since COVID-19 was first discovered in Wuhan, Hubei Province, China, in December 2019, confirmed and suspected cases have been reported in other parts of China and abroad (Wang C. et al., 2020). The outbreak has now reached a pandemic level, with a high global mortality rate (Baud et al., 2020). It has attracted global attention because of its potential for widespread human infection and economic loss. Although the characteristics and pathogenesis of COVID-19 are gradually being revealed, the efficacy and safety of corticosteroid use in patients remain controversial (Russell et al., 2020; Zhong et al., 2020).

Immunological studies have shown that the higher concentration of cytokines and chemokines were detected in patients with COVID-19, and the release of excessive proinflammatory cytokines can promote patients to ARDS, multiple organ dysfunction, and death (Isidori et al., 2020; Shi et al., 2020; Yang J. et al., 2020). Immune suppression is a typical function of corticosteroids (Sehgal and Malhotra, 2019), and treatment with corticosteroids in COVID-19 patients might improve severe clinical symptoms and reduce mortality. Moreover, for coronavirus pneumonia (such as SARS and MERS, caused by SARS-CoV and MERS-CoV, which is similar SARS-CoV-2), corticosteroids are the main anti-inflammatory therapy (Zhong and Zeng, 2003; Arabi et al., 2017), and numerous clinical studies have reported the efficacy of corticosteroids in the treatment of these diseases (Zhao et al., 2003; Chen et al., 2006). Based on the above evidence, it is possible that corticosteroid therapy may be effective in patients with COVID-19.

Some clinical studies also showed that corticosteroid therapy was effective in patients with COVID-19. A retrospective review was conducted by Wang et al. to explore the efficacy of the early use of short-term corticosteroids compared with a control treatment in hospitalized patients with severe COVID-19 pneumonia in Wuhan Union Hospital and reported a remarkable improvement of clinical symptoms and chest computed tomography (CT) findings (Wang et al., 2020a). Moreover, in a cohort study involving 201 patients with confirmed COVID-19 pneumonia, the authors found that the use of methylprednisolone notably reduced the risk of death (Wu C. et al., 2020). Furthermore, China’s “Diagnosis and Treatment Protocol for COVID-19 (Trial Seventh Edition)” also mentions that different doses and treatment courses of methylprednisolone are recommended for general (with high-risk factors for severe COVID-19), severely ill, and critically ill patients with COVID-19 pneumonia to prevent inflammation and reduce exudation (General Office of National Health Commission of People’s Republic of China, 2020). All of these findings seem to indicate the effectiveness of corticosteroids in the treatment of COVID-19.

However, the interim guidelines from the WHO on the clinical management of severe acute respiratory infection when novel coronavirus (SARS-CoV-2) infection is suspected do not advise the use systemic corticosteroids (Organization, 2020); other clinical trials have also indicated that the use of corticosteroids in patients with COVID-19 may not be a good choice. A retrospective, single-center case series including 138 consecutive hospitalized patients conducted by Wang et al. reported no effective outcomes in 44.9% of COVID-19 patients treated with glucocorticoids (Wang D. et al., 2020). Similar results were observed in Kui’s study. The recently published retrospective study by Kui et al. reported no beneficial effect of the use of methylprednisolone on clinical outcomes (Kui et al., 2020). Moreover, previous studies have shown inconclusive clinical evidence of the effect of corticosteroid therapy for viral pneumonia (such as SARS, MERS, and H1N1), and pulse-dose therapy or long-term use of a high-dose corticosteroid in the early stage of disease might be harmful (Stockman et al., 2006; Arabi et al., 2017; Moreno et al., 2018).

Given this background, the effectiveness of corticosteroid therapy for COVID-19 urgently needs to be evaluated. Two recently published articles have reviewed and summarized this issue and reported inadequate evidence to support the routine use of corticosteroids in COVID-19 patients (Li et al., 2020; Veronese et al., 2020). However, one of the two reviews included only four retrospective studies because of its early publication date (Veronese et al., 2020), and the result of the other review was based on disease caused by similar viruses (SARS-CoV and MERS-CoV) but not COVID-19 specifically (Li et al., 2020). Therefore, the results of these published reviews might be inaccurate, and the effects of corticosteroid therapy in COVID-19 patients remain unclear.

In this study, we conducted a systematic review and meta-analysis to evaluate the efficacy and safety of the use of corticosteroids in patients with COVID-19. All the types of literature currently available were included, including randomized controlled trials (RCTs), prospective or retrospective studies, case series, and case reports. The eligible RCTs and cohort studies were used for quantitative synthesis (meta-analysis), and the remaining types of studies were used for qualitative synthesis.

## Methods

The present study was conducted based on the Preferred Reporting Items for Systematic Reviews and Meta-Analyses (PRISMA) guidelines and a previously published protocol (PROSPERO: CRD42020184545) (Moher et al., 2015).

### Search Strategy

The electronic databases PubMed, Cochrane Library, Embase, Web of Science, China National Knowledge Infrastructure (CNKI), WANFANG DATA, and China Science and Technology Journal Database (VIP) were searched for studies. The search terms were “COVID-19 OR 2019 novel coronavirus disease OR SARS-CoV-2 OR 2019-nCoV OR COVID-19 pandemic AND corticosteroid OR glucocorticoid OR steroid OR methylprednisolone OR hydrocortisone OR prednisone.” We manually searched for further articles by tracing the references included in the articles. The database search was run from database inception until 30 July 2020. One reviewer first evaluated the literature to select the studies based on the title and abstract, followed by reading the full text of the remaining reports.

### Study Selection and Outcomes

Studies were selected according to the following inclusion criteria: (1) patients diagnosed with COVID‐19, regardless of severity; (2) patients treated with corticosteroids; (3) patients assessed the efficacy or safety of corticosteroids; (4) because of the scarcity of literature, no restriction on study type or study design (be either a randomized controlled trial, prospective or retrospective study, case series or case report); and (5) published in English and/or Chinese.

The primary outcomes of this study included clinical improvement (recovery and duration of symptoms), mortality, virus clearance time, and adverse events (AEs). The secondary outcomes included utilization of mechanical ventilation, length of intensive care unit (ICU) hospitalization and hospital stay, and discharge data.

### Data Collection and Extraction

Data were independently extracted by two investigators using a standardized data-recording form, and disagreements between authors were discussed with corresponding author. The following information was extracted from the included studies: (1) study characteristics: 1^st^ author, year of publication, study design, study region/country, and sample size; (2) population characteristics: mean/median age, sex, degree of severity, and original comorbidities; (3) corticosteroid treatment: the corticosteroid type, dosage, and duration; and (4) outcomes: primary and secondary outcomes. We applied a Java program called Plot Digitizer (http://plotdigitizer.sourceforge.net/) to convert plotted values into numerical form if adequate information was not provided by the study. The corresponding authors were contacted if the data of interest were not reported.

### Quality Assessment

All eligible studies were assessed by two other authors independently. A third reviewer arbitrated in cases of disagreement. The methodological quality of prospective or retrospective studies was assessed by the New-castle Ottawa Scale (NOS) considering three domains: subject selection, comparability of the study groups, and assessment of outcome (Wells et al.). A NOS score ≥7 was considered high quality. The quality of evidence was assessed with the Grading of Recommendations, Assessment, Development, and Evaluation (GRADE) approach and was graded as high, moderate, low, or very low (Meader et al., 2014).

### Data Synthesis and Statistical Analysis

Data analyses were conducted using STATA 12.0 software. The effect sizes were estimated by risk ratios (RRs) with 95% conﬁdence intervals for dichotomous data and weighted mean diﬀerences (WMDs) with 95% conﬁdence intervals for continuous data. Due to differences in sample characteristics and the small number of studies included, a random effects model was chosen to pool the effect sizes. The *I^2^* statistic was applied to measure heterogeneity among the studies in each analysis, and we considered an *I^2^*>50% as notable heterogeneity. Subgroup analysis was performed according to the severity of disease, including severe only and other. Only cohort studies (corticosteroid group vs control group) were used for quantitative synthesis, and eligible retrospective cohort studies (without control group) and case studies were used for qualitative synthesis. In addition, if the reported results of the included studies were median and range, and the author that be contacted to request the original data cannot be recovered, we would convert the median and range into mean and standard deviation through the data conversion method applied by Hozo et al (Hozo et al., 2005).

## Results

**Figure 1:**
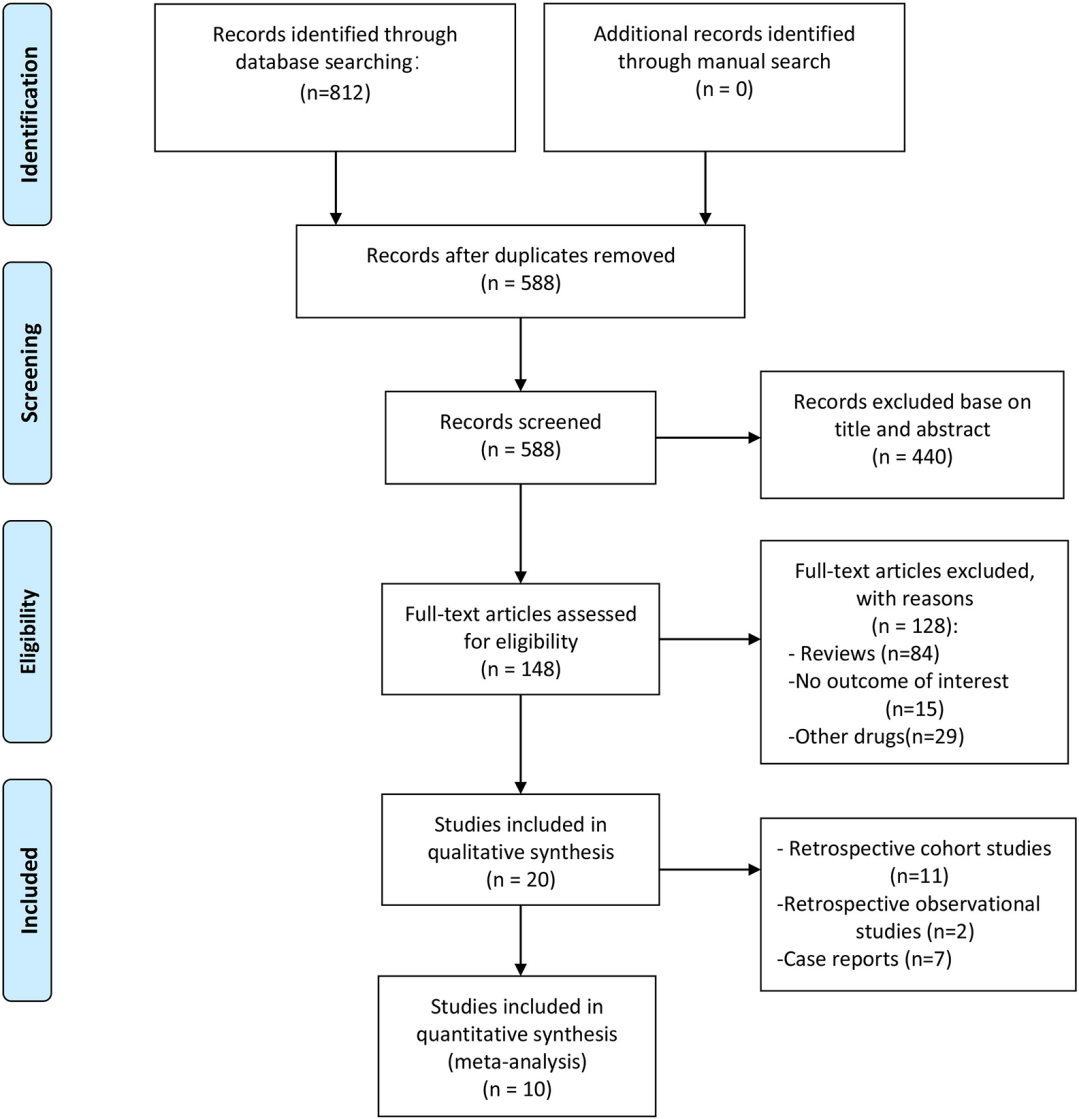
Flow diagram of the study selection and inclusion process.

The initial literature search identified 812 publications. After duplicate publication removal and screening of titles and abstracts, 664 citations were excluded, and 148 studies were selected for a full-text review. Following application of the eligibility criteria, 20 articles with 2840 patients met the inclusion criteria. A summary of the literature search according to the PRISMA flowchart is presented in **Figure 1**.

### Characteristics of the Included Studies

This study included 20 studies for qualitative synthesis. Eleven of these 20 studies were cohort studies (corticosteroid group vs control group) (Fadel et al., 2020; Fang et al., 2020a; Fang et al., 2020b; Ling et al., 2020; Lu et al., 2020; Ni et al., 2020; Wang et al., 2020b; Wu C. et al., 2020; Wu J. et al., 2020; Yang X. et al., 2020; Zha et al., 2020; Zhou F. et al., 2020), and two were retrospective cohort studies (without control group) (Li and Liu, 2020; Zhou W. et al., 2020), while the remaining 7 publications were case studies (Chen et al., 2020; Coyle et al., 2020; Han et al., 2020; Kui et al., 2020; Luo et al., 2020; Zhang et al., 2020; Zheng et al., 2020). In five studies, the publication language was Chinese (Li and Liu, 2020; Luo et al., 2020; Ni et al., 2020; Zhang et al., 2020; Zheng et al., 2020), and in the others it was English. Two study was conducted in America (Coyle et al., 2020; Fadel et al., 2020), and the eighteen other studies were conducted in China. The intervention in the eligible studies was corticosteroid therapy, including methylprednisolone (12 studies), corticosteroid (5 study), glucocorticoids (1 study), methylprednisolone sodium succinate (1 study), steroids (1 study), and prednisone (1 study).

Summaries of the demographic and clinical characteristics of the included cohort studies (corticosteroid group vs control group), retrospective cohort studies (without control group) and case studies are shown in **Tables 1**–**3**, respectively. Summary of the interventions and outcomes of the included cohort studies (corticosteroid group vs control group) and retrospective cohort studies (without control group) are showed in **Tables 4** and **5**, respectively.

**Table 1:** Summary of the demographic and clinical characteristics of the included cohort studies (corticosteroid group vs control group).

Study	Country	Study design	Number of patients (Exp/Ctr)	Median Age, y (Exp/Ctr)	Sex (male%) (Exp/Ctr)	Severe (%) (Exp/Ctr)	Original comorbidities (%)	
							HTN (Exp/Ctr)	DM (Exp/Ctr)
Fadel et al., 2020	America	Cohort	213(132/81)	61/64	51.5/50.6	NR	72.7/76.5	51.5/45.7
Fang et al., 2020a	China	Cohort	55(9/46)	40.2/39.9 (mean)	55.6/47.8	0/0	11.1/8.7	11.1/6.5
Fang et al., 2020b	China	Cohort	23(16/7)	60.6/54.3	75/71.4	100/100	50/28.6	25/0
Ling et al., 2020	China	Cohort	66(5/61)	51/41	20/44.3	0/0	NR	NR
Lu et al., 2020	China	Cohort	244(151/93)	64/59	55.0/48.4	100/100	40.4/36.6	22.5/10.8
Ni et al., 2020	China	Cohort	72 (51/21)	52/46	56.9/57.1	74.5/33.3	37.3/0	5.9/9.5
Wang et al., 2020b	China	Cohort	46(26/20)	54/53	61.5/50.0	100/100	30.8/30	11.5/5
Wu C. et al., 2020	China	Cohort	84(50/34)	58.5 for total	71.6 for total	100/100	27.4 for total	19.0 for total
Wu J. et al., 2020	China	Cohort	1763(690/1073)	63/60 for severe 68/67 for critical	54.8/44 for severe 59.1/58.9 for critical	100/100	23/23.6 for severe 42.8/43.3 for critical	13.4/11.2 for severe 22.0/23.3 for critical
Yang X. et al., 2020	China	Cohort	52 (30/22)	1.9 for survivors; 64.6 for nonsurvivors	0 for survivors; 66 for nonsurvivors	100/100	0 for survivors; 0 for nonsurvivors;	10 for survivors; 22 for nonsurvivors;
Zha et al., 2020	China	Cohort	31(11/20)	53/37	73/60	100/100	18/25	9/0
Zhou F. et al., 2020	China	Cohort	191(57/134)	52 for survivors; 69 for nonsurvivors	59 for survivors; 70 for nonsurvivors	NR	23 for survivors; 48 for nonsurvivors	14 for survivors; 31 for nonsurvivors

DM, diabetes mellitus; HTN, hypertension; Exp, experiment group; Ct, control group; NR, not report.

**Table 2:** Retrospective observational studies involving the use of corticosteroids in patients with COVID-19 (without control group).

Study	Country	Study design	Number of patients	Median Age, y	Sex (male%)	Severe (%)	HTN	DM
Zhou W. et al. 2020	China	Retrospective	15	61.7	66.7	100	40	46.7
Li et al., 2020	Treated with methylprednisolone sodium succinate time							
Aggravation<48 h	China	Retrospective	87	NR	NR	100	NR	NR
Aggravation >48h	China	Retrospective	25	NR	NR	100	NR	NR

DM, diabetes mellitus; HTN, hypertension; NR, not reported.

**Table 3:** Case studies involving the use of corticosteroids in patients with COVID-19.

Study	Country	Age	Sex	Comorbidities	Symptoms	Patchy ground-glass opacities	Mechanical ventilation	Intervention	Death	Clinical recovery	Discharged
Case series											
Kui et al., 2020	China	55 (Median)	61% (M)	HTN, DM, COPD, et al	Fever, cough, myalgias, expectoration, etc	Yes	Yes	Methylprednisolone: 30– 80 mg/day, IV	NR	No significantly benefits	NR
Case reports											
Chen et al., 2020 (case 1)	China	62	F	NR	NR	NR	Yes	Methylprednisolone: 40 mg/12 h	No	Yes	Yes
Chen et al., 2020 (case 2)	China	NR	M	NR	NR	NR	Yes	Methylprednisolone: 40 mg/12 h	No	Yes	NR
Coyle et al., 2020	America	57	M	Myocarditis and ARDS	Fever, cough, myalgias, inappetence, nausea, diarrhea, and SOB	Yes	Yes	Methylprednisolone: 500 mg/day IV	No	Yes	Yes
Han et al., 2020 (case 1)	China	47	F	SLE	Cough, nasal congestion, and runny nose	Yes	No	Oral prednisone: 7.5 mg/d	No	Yes	Yes
Han et al., 2020 (case 2)	China	81	M	PC and CHD	Cough, anorexia, fever, and SOB	Yes	No	Steroids: 80 mg/d	No	Yes	NR
Luo et al., 2020	China	43	M	Cirrhosis and HTN	Fever, cough, headache, pharyngalgia, cold intolerance	Yes	No	Methylprednisolone: 40–80 mg	No	Yes	NR
Zhang et al., 2020	China	55	F	DM	Fever, cough, and diarrhea	Yes	Yes	Methylprednisolone: 40 mg/12 h, iv gtt	No	Yes	Yes
Zheng et al., 2020	China	42	M	No	Fever, cough, myalgias, chest tightness, and SOB	Yes	NR	Methylprednisolone: 40 mg once per day	No	Yes	NR

DM, diabetes mellitus; HTN, hypertension; SLE, systemic lupus erythematosus; PC, prostate cancer; COPD, chronic obstructive pulmonary disease; SOB, shortness of breath; NR, not report.

**Table 4:** Summary of the interventions and outcomes of the included cohort studies (corticosteroid group vs control group).

Study	Intervention	Primary outcomes			Secondary outcomes
		Clinical improvement (Exp/Ctr)	Mortality (Exp/Ctr)	VCT (Exp/Ctr)	
Fadel et al., 2020	MP: 0.5–1 mg/kg/day in 2 divided doses for 3 days (non-ICU), 3–7 days (ICU), IV	NR	13.6/26.3	NR	Significant reduction in median hospital length of stay in the early corticosteroid group: 5 (3–7) vs 8 (5–14) days.
Fang et al., 2020a	MP: 237.5 mg/day ×7 day, oral	NR	NR	VCT: 17.6±4.9/18.7±7.7	NR
Fang et al., 2020b	MP: 250 mg/day ×4.5 day, IV	NR	NR	VCT: 18.8±5.3/18.3±4.2	NR
Ling et al., 2020	Corticosteroid	NR	NR	VCT: 15 (9.8–16.8)/8 (6.0–11.0) (Corticosteroid group was longer than non-corticosteroid group).	NR
Lu et al., 2020	Corticosteroid: 80~100mg/d	NR	Corticosteroid treatment was not significantly associated with 28-day mortality.	NR	NR
Ni et al., 2020	MP: 0.75~1.5mg/kg/d	NR	NR	VCT: 15 (13–20)/14 (12–20) (NSD)	Transient hyperglycemia (94.1%), hypokalemia (9.8%), skin eruption (9.8%), and increased blood pressure (7.8%).
Wang et al., 2020b	MP: 1–2 mg/kg/day for 5–7 days, IV	NR	7.7/5.0 (NSD)	The MP therapy was associated with a faster decrease in CRP and IL-6.	The MP therapy was associated with a shorted length of ICU hospitalization and hospital stay. Length of ICU hospitalization: 8 (6–9)/15 (9–19) Length of hospitalization: 14 (11–16)/22 (18–26)
Wu C. et al., 2020	MP	NR	46.0/61.7	NR	NR
Wu J. et al., 2020	Equivalent to 40 mg MP daily	NR	22.2/3.7	NR	NR
Yang X. et al., 2020	Glucocorticoids	NR	53.3/72.7	NR	NR
Zha et al., 2020	MP: 40 mg once or twice per day × 5 days	MP group showed no different in time to clinical improvement of symptoms compared with control group. Recovery: 100%/75% (NSD) Duration of symptoms: 8 (5–12)/6.5 (4–9.25) (NSD)	No deaths	VCT: 15 (14–16)/14 (11–17) (NSD)	Hospital length of stay were similar in both groups. Length of hospitalization: 20 (18–21)/17 (15.5–9.5) (NSD)
Zhou F. et al., 2020	Systematic corticosteroid	NR	45.6/20.9	NR	NR

MP, methylprednisolone; Exp, experiment group; Ctr, control group; CRP, C-reactive protein; IL-6: interleukin-6; VCT, virus clearance time; NSD, no significant difference; NR, no report.

**Table 5:** Summary of the interventions and outcomes of the included retrospective studies (without control group).

Study	Intervention	Conjunct therapy	Primary outcomes			Secondary outcomes
			Clinical improvement	Mortality (%)	Laboratory tests	
Zhou W. et al., 2020	Corticosteroids, 400.0 mg/day; average, 9.5 days	Noninvasive oxygen therapy and antibiotics and/or antiviral agents	NR	46.7 (7/15)	NR	NR
Li et al., 2020	Treated with methylprednisolone sodium succinate time					
MT < 48 h	Methylprednisolone sodium succinate: 40 mg/day × 3~4 day	Antibiotics, antiviral agents, and standard care	The effective rate reached (recovery): 96.55%	3.4 (3/87)	NR	NR
MT > 48 h	Methylprednisolone sodium succinate: 40 mg/day × 3~4 day	Antibiotics, antiviral agents, and standard care	The effective rate reached (recovery): 20%	80 (20/25)	NR	NR

NR, not reported.

### Methodological Quality of the Included Studies

The meta-analysis included only cohort studies (corticosteroid group vs control group). One of the cohort studies (corticosteroid group vs control group) was excluded because it did not report numerical data of interest (Lu et al., 2020). Finally, ten cohort studies (corticosteroid group vs control group) were included in the quantitative synthesis. The NOS scores of the ten cohort studies ranged from 6 to 9, indicating that most of the eligible cohort studies had a low risk of bias (**Table 6**).

**Table 6:** Risk of bias summary: the review authors’ judgments about each risk-of-bias item for each included cohort study (corticosteroid group vs control group).

Study	Selection	Comparability	Outcome	NOS score	Risk of bias
Fadel et al., 2020	3	2	3	8	L
Fang et al., 2020a	3	2	3	8	L
Fang et al., 2020b	3	2	3	8	L
Ling et al., 2020	3	2	3	8	L
Ni et al., 2020	3	2	3	8	L
Wang et al., 2020b	3	2	3	8	L
Wu C. et al., 2020	3	2	3	8	L
Wu J. et al., 2020	3	1	3	7	L
Yang X. et al., 2020	3	0	3	6	M
Zha et al., 2020	4	2	3	9	L
Zhou F. et al., 2020	3	0	3	6	M

The summary risk of bias was determined as low (NOS scores≥7), moderate (4≤NOS scores≤6), and high (NOS scores≤3).

NOS, New-Castle Ottawa scale; L, low risk; M, moderate risk; H, high risk.

### Outcomes

Summary of the meta-analysis results are shown in **Table 7**.

**Table 7:** Summary of the meta-analysis results.

Outcomes	Effect Size Summary(RR/WMD)	95% CI	Z	*p*-value for Z	Heterogeneity		
					Heterogeneity statistic	I-squared	*P*
Clinical improvement							
Recovery	1.30	0.98, 1.72	1.82	0.068	–	–	–
Duration of symptoms	1.69	−0.24, 3.62	1.72	0.086	–	–	–
Mortality	1.59	0.69, 3.66	1.09	0.277	76.77	93.5%	0.000
Severe only	1.80	0.51, 6.33	0.92	0.359	62.02	95.2%	0.000
Other	1.27	0.43, 3.78	0.43	0.669	9.39	89.3%	0.002
Virus clearance time	1.01	−0.91, 2.92	1.03	0.302	9.29	57.0%	0.054
Severe only	0.85	−1.38, 3.08	0.75	0.455	0.04	0.0%	0.840
Other	1.43	−2.19, 5.05	0.78	0.438	9.03	77.8%	0.011
Mechanical ventilation	0.35	0.10, 1.18	1.70	0.089	–	–	–
Length of hospitalization	−3.17	−7.37, 1.04	1.48	0.140	97.72	98.0%	0.000
Length of ICU hospitalization	−6.50	−7.63, −5.37	11.24	0.000	–	–	–

#### Clinical Improvement

Among the cohort studies (corticosteroid group vs control group), only one study that compared 31 patients (11 in the corticosteroid treatment group and 20 in the control group) was included in the meta-analysis (Zha et al., 2020). Corticosteroid treatment did not significantly shorten the duration of symptoms (WMD = 1.69, 95% CI [−0.24 to 3.62]) (**Table 7**). The meta-analysis revealed a tendency of significantly higher recovery rate in the corticosteroid group than in the control group (RR = 1.30, 95% CI [0.98, 1.72]) (**Table 7**).

One out of two included retrospective cohort studies (without control group) reported the outcome of clinical improvement (Li and Liu, 2020). Li and colleagues retrospectively studied the clinical data of 112 cases treated with low-dose methylprednisolone sodium succinate when mild and noncritical cases became severe and critical and reported that the recovery rates from aggravation of COVID-19 within 48 h and after 48 h were 96.55% and 20%, respectively (**Table 5****)**. Among the included case studies, only one study reported that there was no beneficial effect of the use of methylprednisolone on clinical improvement (Kui et al., 2020). The remaining studies showed that all patients had recovered from COVID-19 at the time of publication **(****Table 3****)**.

#### Mortality

**Figure 2:**
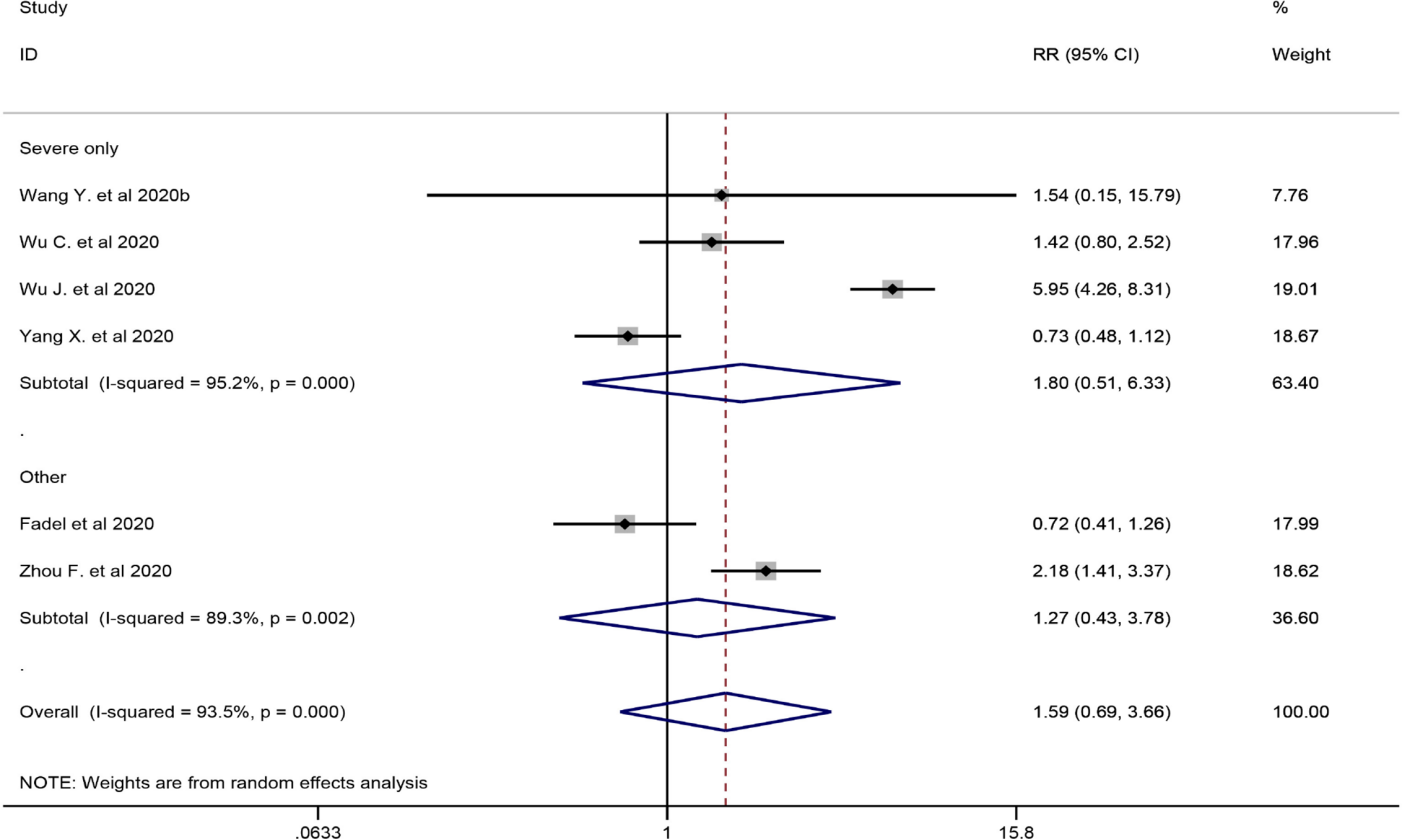
Forest plot of RRs and 95% CIs for subgroup analysis of mortality (severe only vs other).

Six studies involving 2349 participants were included in the meta-analysis to explore the impact of corticosteroid treatment on mortality (Fadel et al., 2020; Wang et al., 2020b; Wu C. et al., 2020; Wu J. et al., 2020; Yang X. et al., 2020; Zhou F. et al., 2020). The results of the pooled analysis indicated that corticosteroids did not significantly reduce mortality (RR = 1.59, 95% CI [0.69, 3.66], *I^2^* = 93.5%) (**Figure 2**). To address heterogeneity, we performed subgroup analysis. The results of the subgroup analysis showed no significant decrease in mortality in the severe only group (RR = 1.80, 95% CI [0.51, 6.33], *I^2^* = 95.2%) and the other group (RR = 1.27, 95% CI [0.43, 3.78]) **(****Figure 2****)**. The eligible cohort studies (corticosteroid group vs control group) that were not included in the quantitative synthesis demonstrated that corticosteroid therapy was not significantly associated with 28-day mortality (Lu et al., 2020).

Both included retrospective cohort studies (without control group) reported the outcome of mortality (Li and Liu, 2020; Zhou W. et al., 2020), and all patients involved in the two eligible studies had severe COVID-19. Zhou et al evaluated the efficacy of corticosteroid treatment in 15 Chinese patients with COVID-19 and reported that death had occurred in 46.7% of the population at the time of publication. Li et al. (2020) reported that the mortality of aggravation of COVID-19 within 48 h and after 48 h was 3.4% and 80%, respectively (**Table 5****)**. No deaths were reported in the included case studies; however, one study did not report mortality in patients using corticosteroids **(****Table 3****)** (Kui et al., 2020).

#### Virus Clearance Time

**Figure 3:**
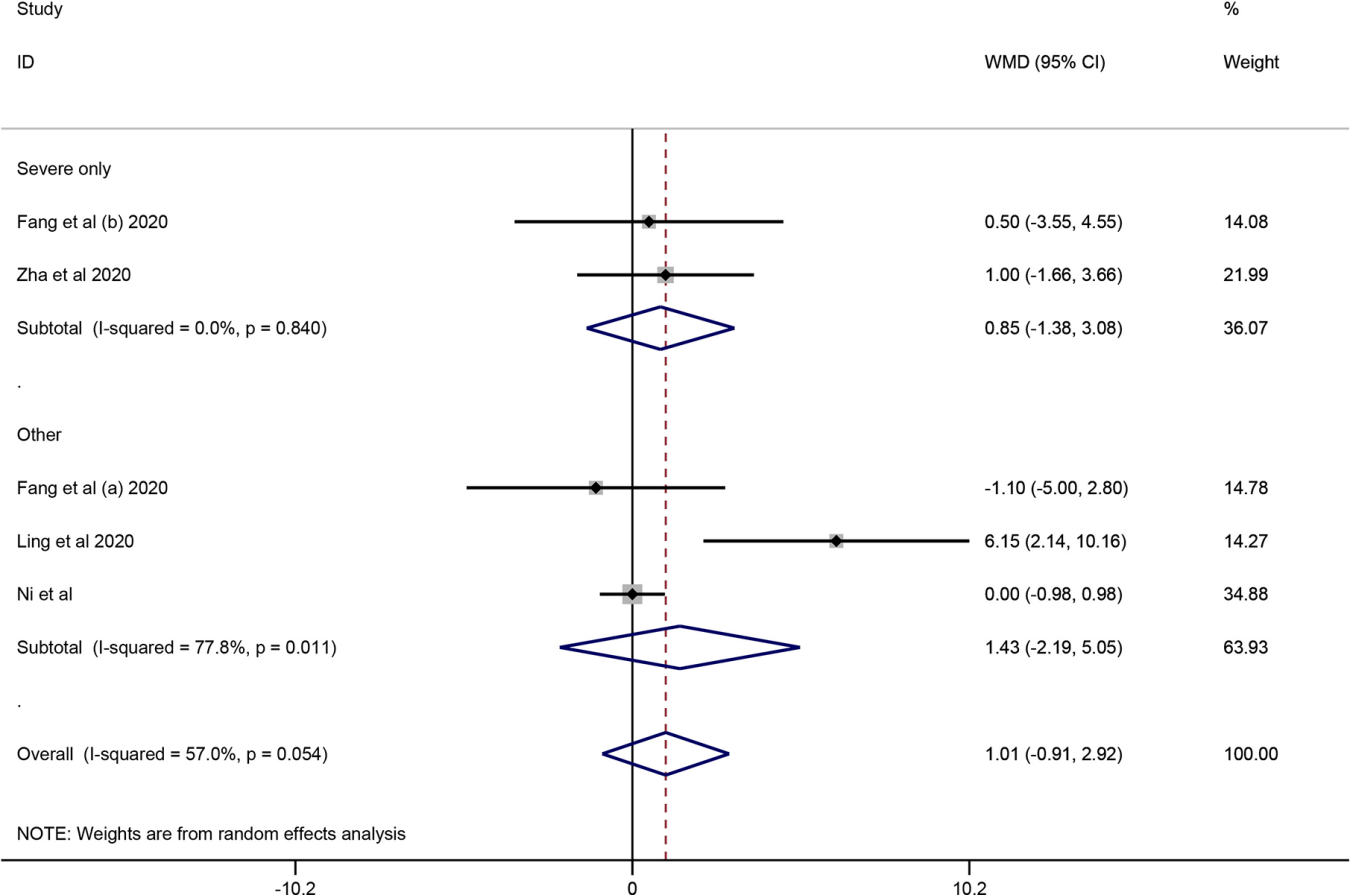
Forest plot of WMDs and 95% CIs for subgroup analysis of virus clearance time (severe only vs other).

Four cohort studies including 247 subjects were included in the meta-analysis to explore the impact of corticosteroid treatment on virus clearance time (Fang et al., 2020a; Fang et al., 2020b; Ling et al., 2020; Ni et al., 2020; Zha et al., 2020). One out of the four studies compared the effects of corticosteroid treatment in the general group and severe group relative to the control group (Fang et al., 2020a; Fang et al., 2020b). Therefore, five trials were included in the quantitative synthesis. The pooled analysis data showed that the virus clearance time in the corticosteroid group was not shorter than that in the control group (WMD = 1.01, 95% CI [−0.91 to 2.92], *I^2^* = 57%) (**Figure 3**). As described in the mortality section, we performed a subgroup analysis. The subgroup analysis showed that corticosteroid treatment did not notably reduce virus clearance time regardless of severe only (WMD = 0.85, 95% CI [−1.38 to 3.08], *I^2^* = 0%) or other group (WMD = 1.43, 95% CI [−2.19 to 5.05], *I^2^* = 77.8%) (**Figure 3****)**.

All the included retrospective cohort studies (without control group) and case studies did not report virus clearance time in patients with COVID-19 after corticosteroid treatment.

#### Mechanical Ventilation

One cohort study involving 46 participants was included in the meta-analysis (Wang et al., 2020b). The results of the meta-analysis indicated that the use of mechanical ventilation was not different between the corticosteroid group and the control group (RR = 0.35, 95% CI [0.10, 1.18]) (**Table 7**). The use of mechanical ventilation in patients treated with corticosteroids was not clearly described in the included retrospective cohort studies (without control group). Among the included case studies, four reported that patients required mechanical ventilation (Chen et al., 2020; Coyle et al., 2020; Kui et al., 2020; Zhang et al., 2020), and two reported that patients did not require mechanical ventilation (Han et al., 2020; Luo et al., 2020). The remaining one study did not report the specific use of mechanical ventilation (Zheng et al., 2020).

#### Lengths of Hospitalization and ICU Hospitalization

**Figure 4:**
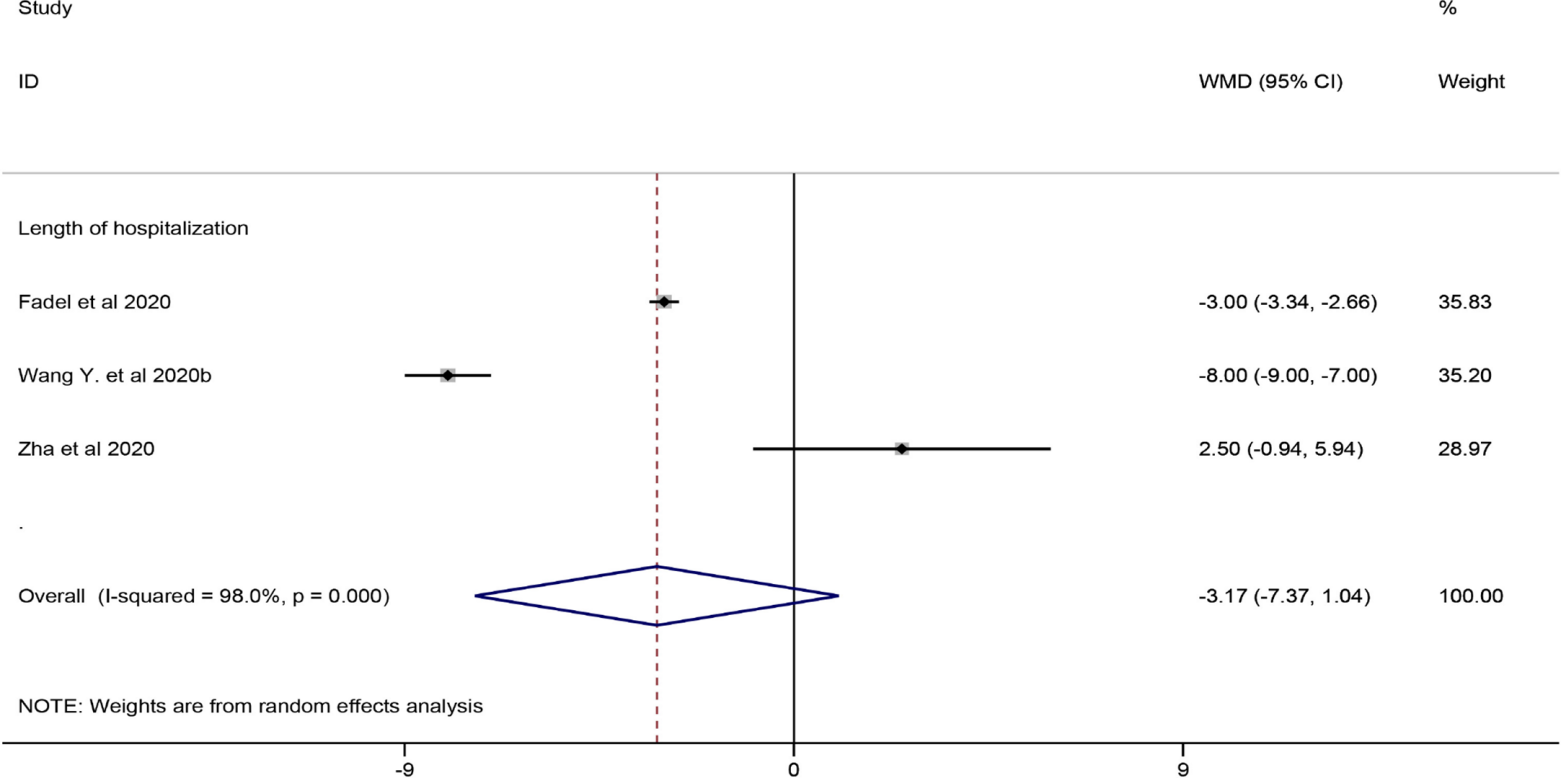
Forest plot of WMDs and 95% CIs for length of hospitalization hospitalization (corticosteroid group vs control group).

Among the eligible studies, three studies involving 290 subjects were included in the meta-analysis of length of hospitalization (Fadel et al., 2020; Wang et al., 2020b; Zha et al., 2020), and one involving 46 participants was included in the meta-analysis of length of ICU hospitalization (Wang et al., 2020b). The pooled results showed that corticosteroid treatment was not associated with a shortened length of hospitalization (WMD = −3.17, 95% CI [−7.37, 1.04]), whereas it remarkably shortened the length of ICU hospitalization (WMD = −6.50, 95% CI [−7.63 to −5.37]) (**Figure 4** and **Table 7**).

#### Safety

Only one included cohort study (corticosteroid group vs control group) reported AEs associated with corticosteroid therapy (Ni et al., 2020). The study reported that among the 51 patients treated with corticosteroids, 94.1% had transient hyperglycemia, 9.8% had hypokalemia, 9.8% had skin eruption, and 7.8% had increased blood pressure, but most of the AEs were mild and controllable. None of the included retrospective studies reported AEs in patients with COVID-19 after corticosteroid treatment.

### Publication Bias

In this analysis, there was no publication bias on Egger test for mortality (*p* = 0.464) and virus clearance time (*p* = 0.174). The results of sensitive-analysis for mortality and virus clearance time see **Supplementary Materials**.

### GRADE Assessment

The GRADE approach was used to evaluate the overall evidence. The quality of evidence is shown in **Table 8**. The quality of evidence of clinical improvement (recovery and duration of symptoms), VCT (severe only and other), mechanical ventilation and length of ICU hospitalization was very low, while the quality of evidence of mortality-severe only, mortality-other, and length of hospitalization was low.

**Table 8:** GRADE summary.

The quality of evidence				
Outcomes	Relative effect(95% CI)	*P*-Value	No of Participants(studies)	Quality of the evidence(GRADE)
**Clinical improvement**				
**Recovery**	**RR 1.30** (0.98 to 1.72)	0.068	31 (1 study)	⊕⊝⊝⊝ **very low**^1,2^
**Duration of symptoms**	**RR 1.69** (−0.24 to 3.62)	0.086	31 (1 study)	⊕⊝⊝⊝ **very low^2^**
**Mortality**				
**Mortality - Severe only**	**RR 1.80** (0.51 to 6.33)	0.359	1945 (4 studies)	⊕⊕⊝⊝ **low^1,3^**
**Mortality – Other**	**RR 1.27** (0.43 to 3.78)	0.669	1976 (2 study)	⊕⊕⊝⊝ **low**^1,3^
**Virus clearance time (VCT)**				
**VCT- Severe only**	**RR 0.85** (−1.38 to 3.08)	0.445	54 (2 studies)	⊕⊝⊝⊝ **very low^2^**
**VCT- Other**	**RR 1.43** (−2.19 to 5.05)	0.438	193 (3 studies)	⊕⊝⊝⊝ **very low**^3^
**Mechanical ventilation**	**RR 0.35** (0.10 to 1.18)	0.089	46 (1 study)	⊕⊝⊝⊝ **very low^1,2^**
**Length of hospitalization**	**RR** −**3.17** (−7.37 to 1.04)	0.140	290 (3 study)	⊕⊕⊝⊝ **low^3^**
**Length of ICU hospitalization**	**RR** −**6.50** (−7.63 to −5.371.18)	0.000	46 (1 study)	⊕⊝⊝⊝ **very low^2^**

*The basis for the **assumed risk** (e.g. the median control group risk across studies) is provided in footnotes. The **corresponding risk** (and its 95% confidence interval) is based on the assumed risk in the comparison group and the **relative effect** of the intervention (and its 95% CI).

**CI**, Confidence interval; **RR**, Risk ratio;

GRADE Working Group grades of evidence

**High quality:** Further research is very unlikely to change our confidence in the estimate of effect.

**Moderate quality:** Further research is likely to have an important impact on our confidence in the estimate of effect and may change the estimate.

**Low quality:** Further research is very likely to have an important impact on our confidence in the estimate of effect and is likely to change the estimate.

**Very low quality:** We are very uncertain about the estimate.

^1^Imprecision (Total number of events is very few).

^2^ Imprecision (Few subjects were included).

^3^ Inconsistency (significant heterogeneity).

## Discussion

The main results of this systematic review and meta-analysis are as follows: (1) treatment with corticosteroid in COVID-19 patients did not significantly shorten the duration of symptoms but may promote clinical recovery; (2) Regardless of disease severity, corticosteroid therapy had no effect on mortality in patients with severe COVID-19; (3) corticosteroid therapy did not significantly reduce the virus clearance time in patients with COVID-19, irrespective of severity; (4) corticosteroid therapy in patients with COVID-19 did not affect the need for mechanical ventilation; (5) corticosteroid therapy was associated with a significantly decreased length of ICU hospitalization but had no impact on the length of hospitalization; and (6) corticosteroid therapy was associated with some mild and controllable AEs, including transient hyperglycemia, hypokalemia, skin eruption, and 7.8% had increased blood pressure.

Our results provide some evidence for the effectiveness of corticosteroids. As shown in **Table 4**, the meta-analysis indicated that compared with the control patients, the COVID-19 patients treated with corticosteroids showed a tendency of improved clinical recovery. This result was also supported by the retrospective cohort studies (without control group) and case studies we included. An eligible retrospective cohort studies (without control group) conducted by Li et al reported a high rate (96.55%) of clinical recovery of COVID-19 aggravation within 48 hours (Li and Liu, 2020). Additionally, except for one case study (Kui et al., 2020), all patients in the included case studies achieved clinical recovery. The above results indicated that treatment with corticosteroids in COVID-19 patients may increase the rate of clinical recovery. However, it is worth noting that the rate of clinical recovery of COVID-19 aggravation after 48 hours in Li et al’ study only reached 20%, which might indicate that clinical recovery was associated with corticosteroid administration time. Data from this study suggested that the use of corticosteroids did not reduce the risk of mortality. This is consistent with results of studies on MERS. A retrospective cohort study involving 309 subjects was conducted to investigate the association of corticosteroid therapy with mortality in critically ill patients with MERS and reported that there was no significant association with 90-day mortality (Arabi et al., 2017).

There has been a long debate about whether corticosteroid use might delay viral clearance in patients with viral pneumonia. The function of corticosteroids is to suppress the immune response as well as innate immunity. However, the elimination of the virus requires the involvement of innate immunity at the early stage, and the use of corticosteroids may delay the clearance of the virus (Ai et al., 2020; Isidori et al., 2020). Our results showed that corticosteroid therapy did not increase the virus clearance time regardless of the severity of COVID-19. Nevertheless, in the literature investigating MERS and influenza A (H7N9) viral pneumonia, the administration of corticosteroids was associated with delayed viral clearance (Cao et al., 2016; Arabi et al., 2017; Wang et al., 2020). Whether corticosteroid therapy delays viral clearance in patients with COVID-19 needs further study. Moreover, the utilization of mechanical ventilation was increased after corticosteroid treatment in patients with MERS and influenza (Arabi et al., 2017), while according to our results, corticosteroid therapy was not associated with the need for mechanical ventilation. Regarding the lengths of hospitalization and ICU hospitalization, our results revealed that corticosteroid treatment in patients with COVID-19 was not associated with a shorted length of hospitalization, whereas it remarkably shortened the length of ICU hospitalization. This might be related to the powerful pharmacological effects of the corticosteroid itself. A series of clinical studies demonstrated that low-dose or physiological-dose corticosteroid treatments in patients with septic shock caused by pulmonary infection significantly shortened the length of ICU hospital stay and reversed the progression of shock (Rhodes et al., 2017; Marik, 2018). Therefore, the clinical use of corticosteroids in patients with severe COVID-19 may reduce the length of ICU hospitalization.

In addition to focusing on the efficacy of corticosteroid therapy, its safety also needs to be fully evaluated. A retrospective analysis of corticosteroid therapy for ARDS caused by H1N1 showed that the early administration of corticosteroids resulted in an increased risk of mortality and increased incidence of acquired pneumonia (Brunbuisson et al., 2011). Some studies and reviews also showed that the adverse effects of corticosteroid therapy for coronavirus pneumonia were significantly greater than the therapeutic effects (Griffith et al., 2005; Brunbuisson et al., 2011; Ruan et al., 2014). Furthermore, a study on the treatment of SARS patients with corticosteroids demonstrated that the occurrence of adverse reactions was positively correlated with the dose and duration of corticosteroid use (Stockman et al., 2006). However, of all the literature included in this study, only one cohort study reported AEs of corticosteroid treatment, including transient hyperglycemia, hypokalemia, skin eruption, and increased blood pressure, in patients with COVID-19, and most of them were mild and controllable (Ni et al., 2020). Additional clinical studies are needed to explore the safety of corticosteroid treatment in patients with COVID-19 in the future.

Recently, emerging evidence from the initial clinical trial revealed a clear benefit of dexamethasone in patients with COVID-19 (Horby et al., 2020; Fadel et al., 2020). A large randomized, controlled, open-label (RECOVERY) trial conducted by Horby et al showed that the use of the dexamethasone to hospitalized COVID-19 patients enhanced the clinical outcome (Horby et al., 2020). In this study, 2104 patients were assigned to receive dexamethasone and 4,321 to receive usual care, and by the end, the incidence of death and duration of hospitalization in the dexamethasone group was lower and shorter than that in the usual care group. Similar results were observed in a recently published study that early treatment with methylprednisolone to hospitalized COVID-19 patients (Fadel et al., 2020), and a significant reduction in median hospital length of stay and mortality was observed in the early corticosteroid group. These beneficial effects might result from reducing the excessive inflammatory responses of COVID-19. As mentioned above, COVID-19 infection can causes a massive cytokine release (namely, cytokine storm), and leading to severe complications, such as acute respiratory distress syndrome (ARDS), disseminated thromboembolism, and hypotensive shock, and leads to a high risk of mortality (Isidori et al., 2020). These diseases are mostly driven by immunoinflammatory reactions. Dexamethasone, as a widely available and inexpensive corticosteroid, can attenuate the immune response and improved the clinical outcome in severe cases of COVID-19. However, the timing of corticosteroid use might be critical. COVID-19 can develop from mild to severe disease, characterized by an initial viral infection stage followed by lung inflammation, and then a hyper-inflammatory stage (Abdin et al., 2020; Siddiqi and Mehra, 2020). Early use of corticosteroids may delay the progression of the disease to the high inflammation stage (Fadel et al., 2020). This was consistent with a study included in our paper, which showed that the use of corticosteroid of COVID-19 aggravation after 48 hours significantly reduces the clinical recovery rate (Li et al., 2020). Moreover, the severity of the disease may also affect the outcome of corticosteroids. Some individuals with mild COVID-19 causes a specific adaptive immune response in the early stage (Isidori et al., 2020; Shi et al., 2020). The initial response begins with the recruitment of innate immune factors. Innate immunity at this stage is beneficial in eliminating the virus and preventing disease progression to severe stages (Shi et al., 2020). However, due to extensive immune suppression, the use of corticosteroids in mild patients with COVID-19 may delay the clearance of the virus by suppressing their own innate immunity (Isidori et al., 2020; Shi et al., 2020). Overall, the outcome of administering corticosteroids seems to largely depend on the severity of the disease and the timing of medication. The use of dexamethasone to achieve the optimal therapeutic improvement may be early use in severe patients with COVID-19.

There are some limitations in this study. First, only two included studies were conducted in America, and the remaining studies were conducted in China, and regional differences may impact the results of studies. Second, some outcomes, such as clinical recovery, duration of symptoms, mechanical ventilation and length of ICU hospitalization were measured in only a small number of studies or even just one study, leading to imprecision in the outcomes, thus decreasing the quality of the evidence. Third, considering the lack of literature and relevant data, we did not conduct subgroup analyses of the type and dosage of corticosteroids in this study.

In conclusion, our work complements recent systematic reviews of the efficacy and safety of corticosteroid treatment in patients with COVID-19 and enables, for the first time, quantitative synthesis of the effects of corticosteroid therapy considering cohort studies. The present study indicated that corticosteroid therapy was associated with clinical recovery and a significantly shortened length of ICU hospitalization, but it did not affect the mortality, the utilization of mechanical ventilation and the virus clearance time in COVID-19 patients. Furthermore, treatment with corticosteroids in patients with COVID-19 may cause mild adverse outcomes. The findings in our study do not demonstrate a strong efficacy of corticosteroid therapy, but it appears to improve prognosis and promote clinical recovery in patients with severe COVID-19.

## Author Contributions

ZC and AX take responsibility for the integrity of data and accuracy of its analysis. WC and YL contributed to the conception and design of the study and writing of the manuscript. YL prepared the figures and tables. WC, LC, YC, SS, DX, and XC all contributed substantially to the literature search, data extraction and analysis, data interpretation and quality assessment.

## Funding

This work was supported by National Key Research and Development Project (2020YFC2005700), Key Realm R&D Program of Guangdong Province (2019B030335001), and Medical Scientific Research Foundation of Guangdong Province of China (A2019407).

## Conflict of Interest

The authors declare that the research was conducted in the absence of any commercial or financial relationships that could be construed as a potential conflict of interest.
