# Supplementary figures and images for: Efficacy and Safety of Corticosteroid Treatment in Patients With COVID-19: A Systematic Review and Meta-Analysis
Source: Front Pharmacol. 2020 Sep 9;11:571156. doi: 10.3389/fphar.2020.571156 (PMC7510504; doi:10.3389/fphar.2020.571156)

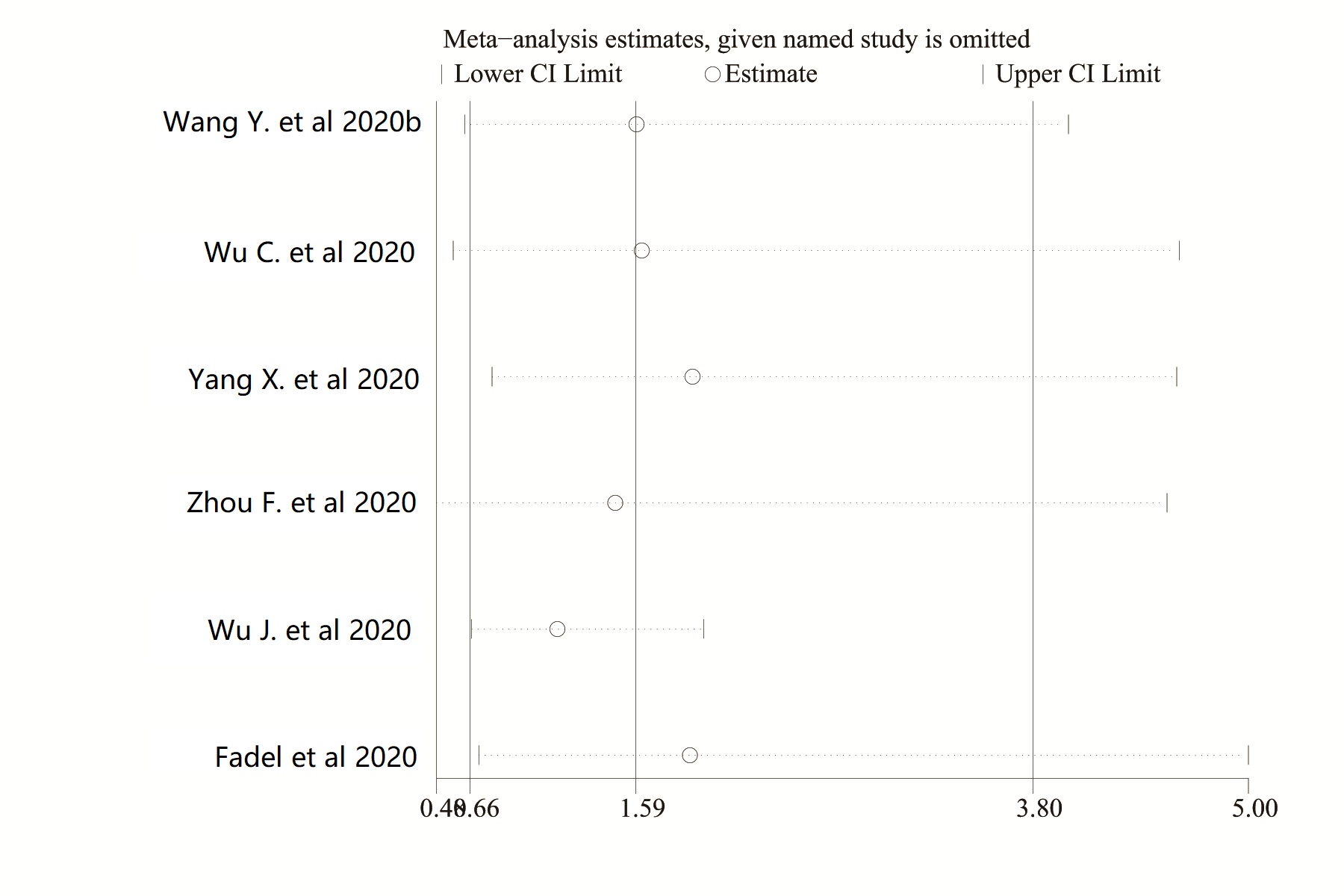

Supplement: Supplementary file 1 [file Image_1.tif]

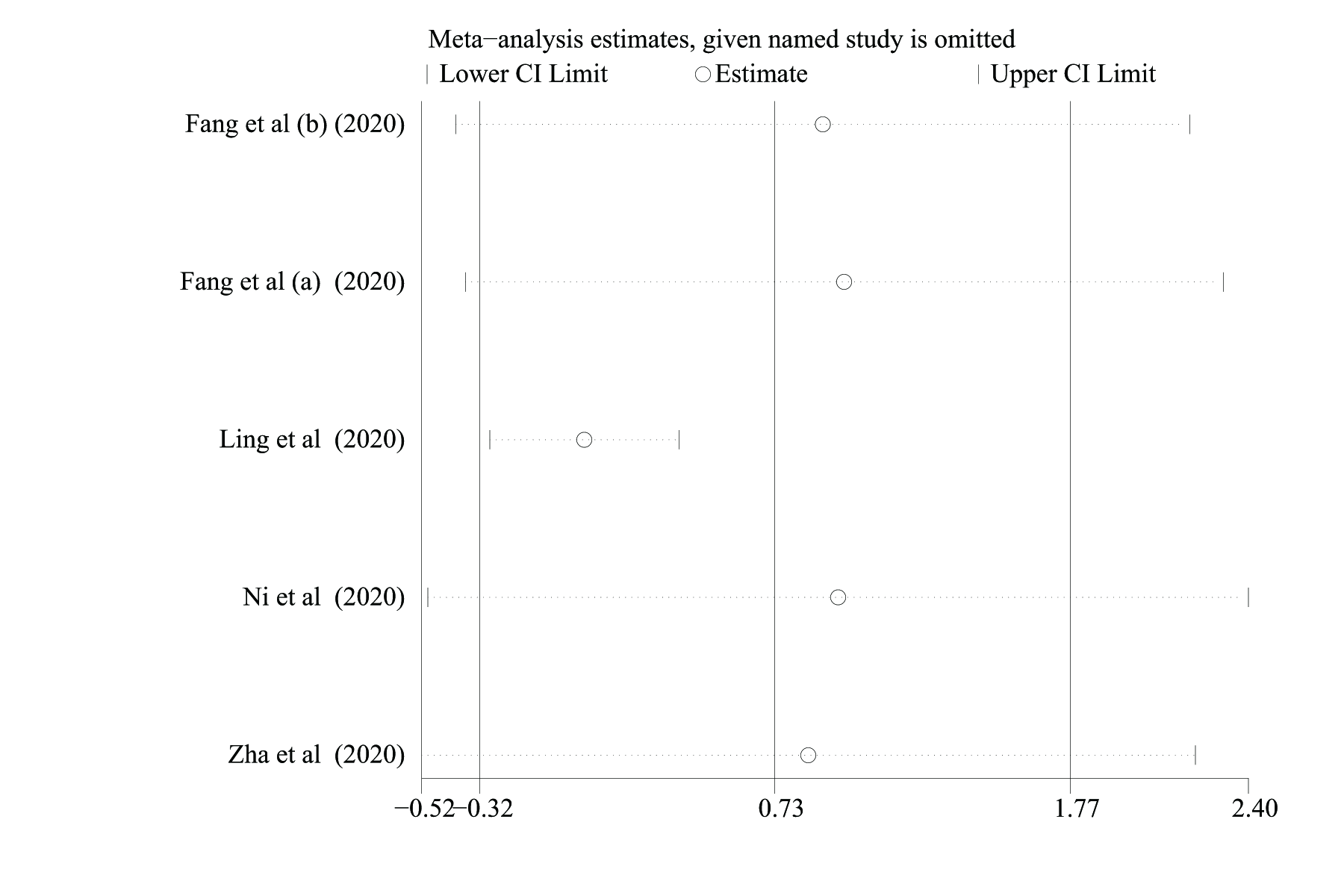

Supplement: Supplementary file 2 [file Image_2.tif]
